# Supplementary material for: Orai1 Boosts SK3 Channel Activation
Source: Cancers (Basel). 2021 Dec 17;13(24):6357. doi: 10.3390/cancers13246357 (PMC8699475; doi:10.3390/cancers13246357)
Supplement: Supplementary file 1 [file cancers-13-06357-s001.zip › cancers-1514554-supplementary.pdf]

Supplementary Material: Orai1 Boosts SK3 Channel Activation

Adéla Tiffner, Valentina Hopl, Romana Schober, Matthias Sallinger, Herwig Grabmayr, Carmen Höglinger, Marc Fahrner, Victoria Lunz, Lena Maltan, Irene Frischauf, Denis Krivic, Rajesh Bhardwaj, Rainer Schindl, Matthias A. Hediger and Isabella Derler

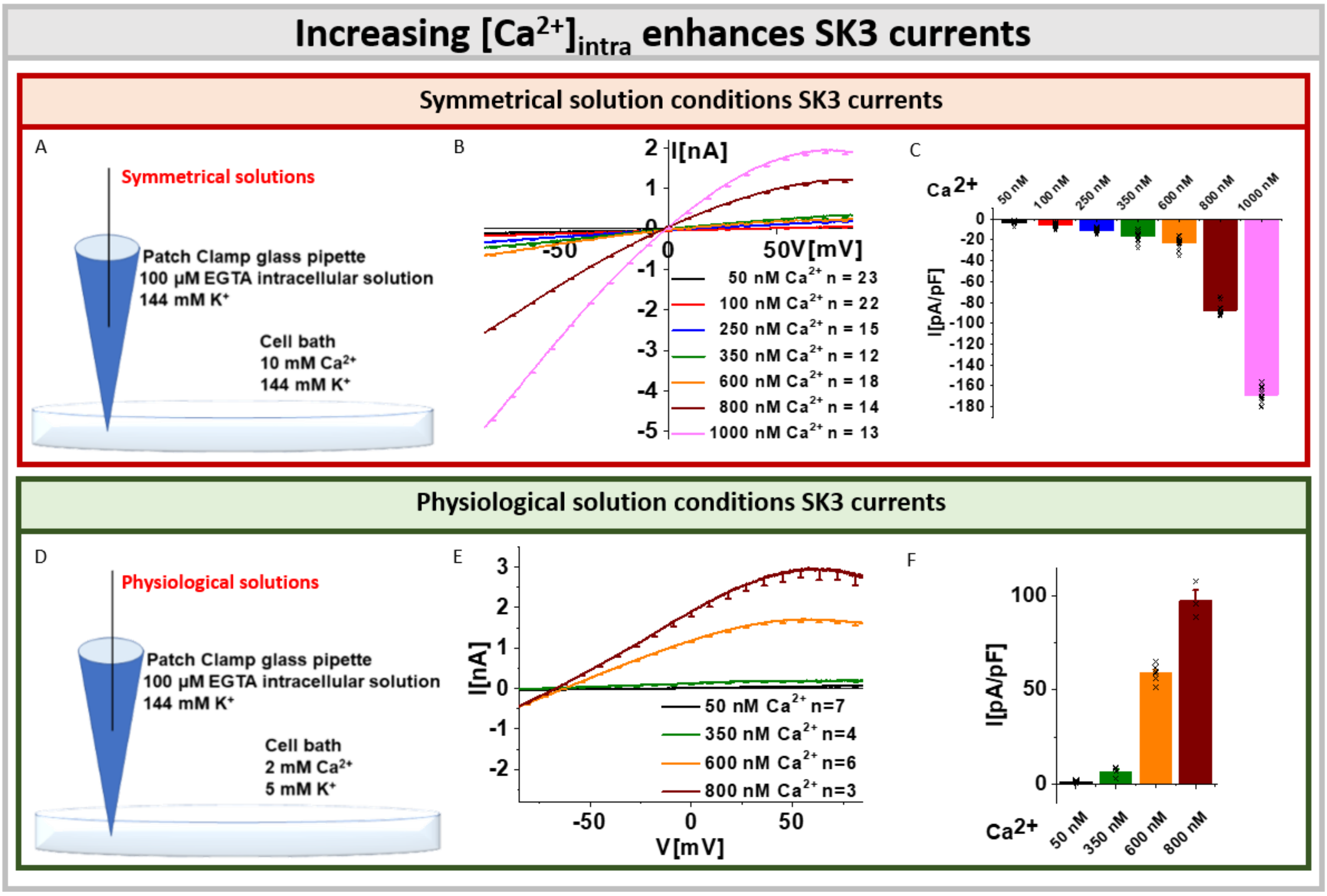

Figure S1. Characterization of SK3 channel. (**A**) Scheme represents the solution conditions used for experiments in (**B**) & (**C**); (**B**) SK3 K^+ current I/V relationships in dependence on the increasing intracellular Ca^{2+} concentration at symmetrical K^+ solution conditions; (**C**) The block diagram corresponds to the maximum currents recorded in (**B**); (**D**) Scheme represents the solution conditions used for experiments in (**E**) & (**F**); (**E**) SK3 K^+ current I/V relationship in dependence on the increasing intracellular Ca^{2+} concentration at physiological K^+ solution conditions; (**F**) The block diagram corresponds to the maximum currents recorded in (**E**); All experiments in Figure S1 were performed in normal HEK 293 cells.

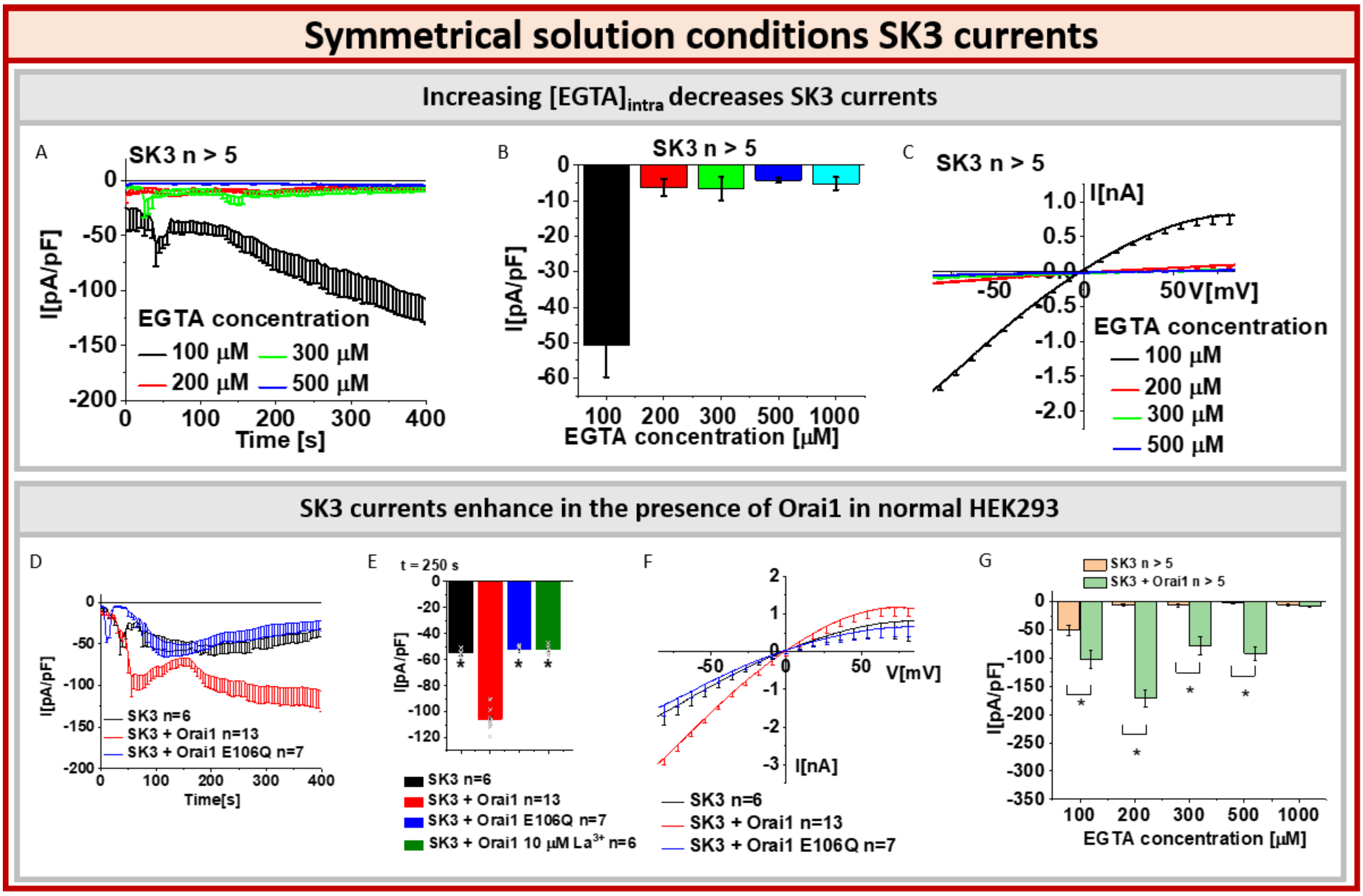

Figure S2. SK3 currents under symmetrical solution conditions. (**A**) Time course of SK3 mediated K^+ currents recorded upon application of different EGTA concentrations in the pipette solution under symmetrical solution conditions; (**B**) Block diagram depicting maximum current densities measured in (**A**); (**C**) I/V relationship corresponding to (**A**); (**D**) Time course of K^+ currents of SK3 channel expressing compared to SK3 and Orai1 or Orai1 E106Q co-expressing HEK 293 cells under symmetrical solution conditions; (**E**) Block diagram with maximum current densities at $t = 250$ s corresponding to (**D**) and Orai1 + SK3 currents upon application of 10 µM La^{3+}. The Mann-Whitney test was used for statistical comparison considering differences statistically significant at $p < 0.05$ as indicated by the asterisks. (**F**) I/V relationship of maximum currents measured in (**D**); (**G**) Maximum K^+ currents of SK3 and SK3 + Orai1 expressing HEK 293 cells in response to different EGTA concentrations in the pipette solution (100, 200, 300, 500, 1000 µM) under symmetrical solution conditions The Mann-Whitney test was used for statistical comparison considering differences statistically significant at $p < 0.05$ as indicated by an asterisk. All experiments in Figure S2 were performed in normal HEK 293 cells.

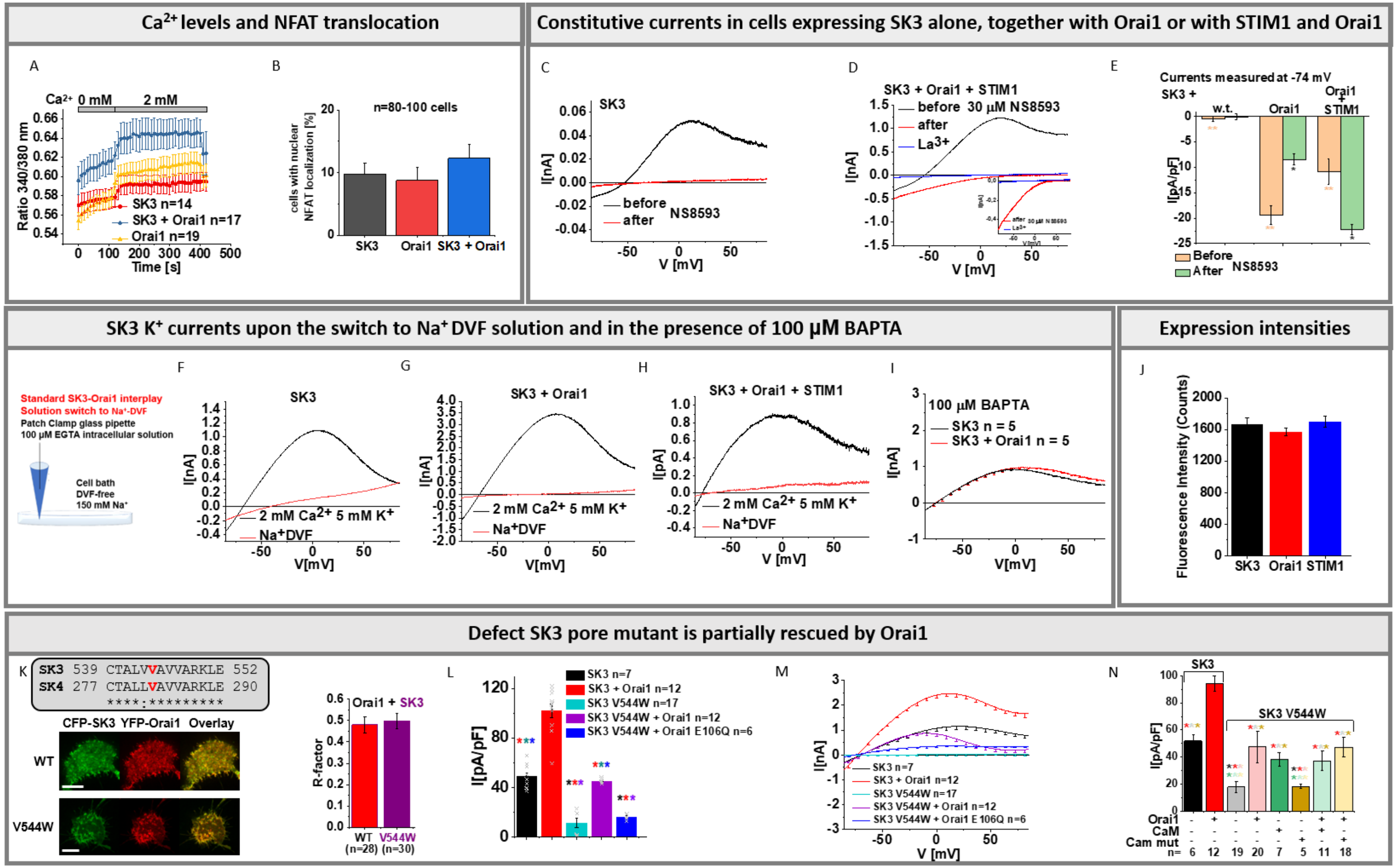

Figure S3. Characterization of SK3 channel K⁺ currents alone compared to the presence of Orai1 or STIM1/Orai1. (**A**) Cytosolic Ca^{2+} concentrations represented by the ratio 340/380 nm of Fura2-AM were monitored initially in a nominally Ca^{2+} free extracellular solution, followed by a solution containing 2 mM Ca^{2+} in STIM1/Orai1 DKO HEK293 cells overexpressing SK3, Orai1 or SK3 + Orai1. (**B**) The percentage of STIM1/Orai1 DKO HEK293 cells that exhibit nuclear NFAT localization determined upon co-expression (NFAT-CFP) with SK3, Orai1 or SK3 + Orai1. (**C**) I/V relationship of SK3 currents under standard STIM1/Orai1 solution conditions before and after application of the blocker NS8593; (**D**) I/V relationship of SK3 + Orai1 + STIM1 mediated K^+ currents recorded under standard STIM1/Orai1 solution conditions before and after addition of the SK channel blocker NS8593 (30 μM). (inset) The remaining current displaying inward rectifying behavior was blocked by La^{3+}. (**E**) The block diagram shows current density before and after application of the SK channel blocker NS8593 for SK3, SK3 + Orai1 and SK3 + Orai1 + STIM1 ($n > 5$) measured at -74 mV. The Mann-Whitney test was used for statistical comparison considering differences statistically significant at $p < 0.05$ as indicated by asterisks of the corresponding color. (**F-H**) I/V relationship of SK3 (**D**), SK3 + Orai1 (e), SK3 + Orai1 + STIM1 (f) before and after the switch to Na^+ -divalent free solution as indicated by the scheme on the left. (**I**) I/V relationship of SK3 compared to SK3 + Orai1 under physiological solution conditions in the presence of 100μM BAPTA instead of 100 μM EGTA intracellularly. (**J**) Expression levels represented by fluorescence intensities of overexpressed CFP-SK3, YFP-Orai1 and Cherry-STIM1. (**K**) Sequence alignment of SK3 and SK4 channel pore region with the narrowest

part highlighted in red (V544 in SK3 and V282 in SK4); Co-localization studies in STIM1/Orai1 DKO HEK 293 cells performed with a pixel-by-pixel analysis and the corresponding block diagram showing the comparison of YFP-Orai1 with CFP-SK3 and YFP-Orai1 with CFP-SK3 V544W (scale bar: 10 μm); (**L**) Block diagram with maximum current densities of the pore mutant SK3 V544W, SK3 V544W + Orai1 and SK3 V544W + Orai1 E106Q compared to SK3 and SK3 + Orai1. The Mann-Whitney test was used for statistical comparison considering differences statistically significant at $p < 0.05$ as indicated by asterisks of the corresponding color; (**M**) The I/V relationship of maximum currents corresponding to (**L**). (**N**) Block diagram with maximum current densities of the pore mutant SK3 V544W in the absence or presence of Orai1, CaM and/or CaM_{MUT}. The Mann-Whitney test was used for statistical comparison considering differences statistically significant at $p < 0.05$ as indicated by asterisks of the corresponding color. Experiments in (**C**-**E**) were performed in normal HEK293 cells while the other experiments in Figure S3 (**A**-**B** and **F**–**N**) were performed in STIM1/Orai1 DKO HEK 293 cells.

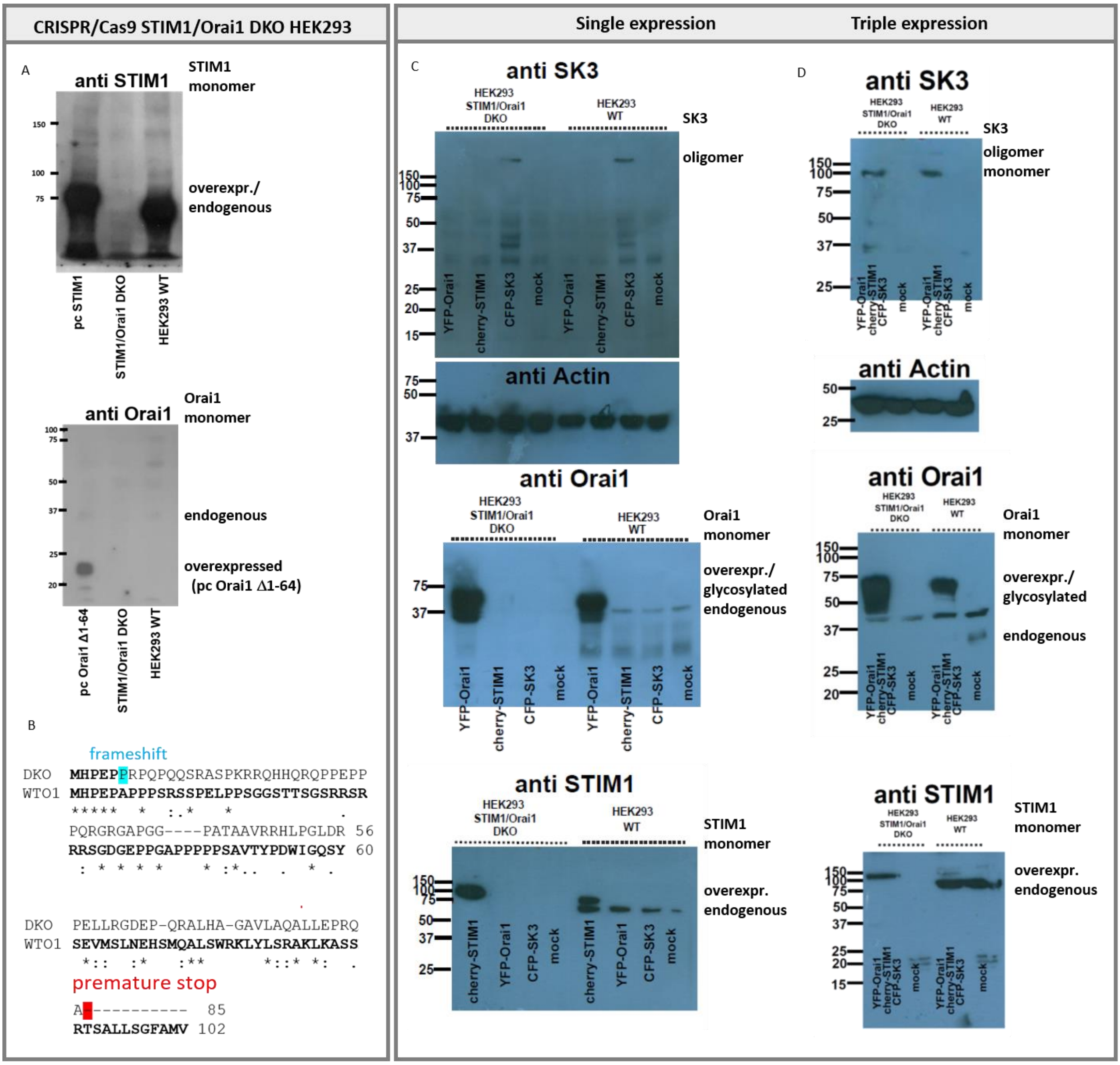

Figure S4. Expression of SK3, Orai1 and STIM1. (**A**) Immunodetection of western blots from cell lysates of wild-type HEK 293 cells and of STIM1/Orai1 DKO HEK 293 cells. Top: anti STIM1 antibody. Bottom: anti Orai1 antibody. Transient overexpression of STIM1-His6 and Orai1 Δ1-64, respectively, served as a positive control in both cell lines. (**B**) gDNA analysis of HEK DKO cell clones. Amino acid alignment of wild-type Orai1 with sequences obtained from gDNA of STIM1/Orai1 DKO cells. The sequence shows a frameshift around aa6 leading to premature termination of Orai1 protein expression. (**C**) (**D**) Western blots of wild-type and S1/O1 DKO HEK 293 cells upon overexpression of either SK3, Orai1 or STIM1 (**C**) or co-expressing SK3, Orai1 and STIM1 (**D**) showing the expression of SK3, Orai1 or STIM1 detected with suitable antibodies. The individual bands of endogenous and/or overexpressed proteins of interest are labeled accordingly to their size (endogenous: SK3: ~81,4 kDa, Orai1: ~33,1 kDa, STIM1: ~75,35 kDa; corresponding labelled proteins: SK3: ~108,4 kDa, Orai1: ~60,1 kDa, STIM1: ~102,4 kDa).

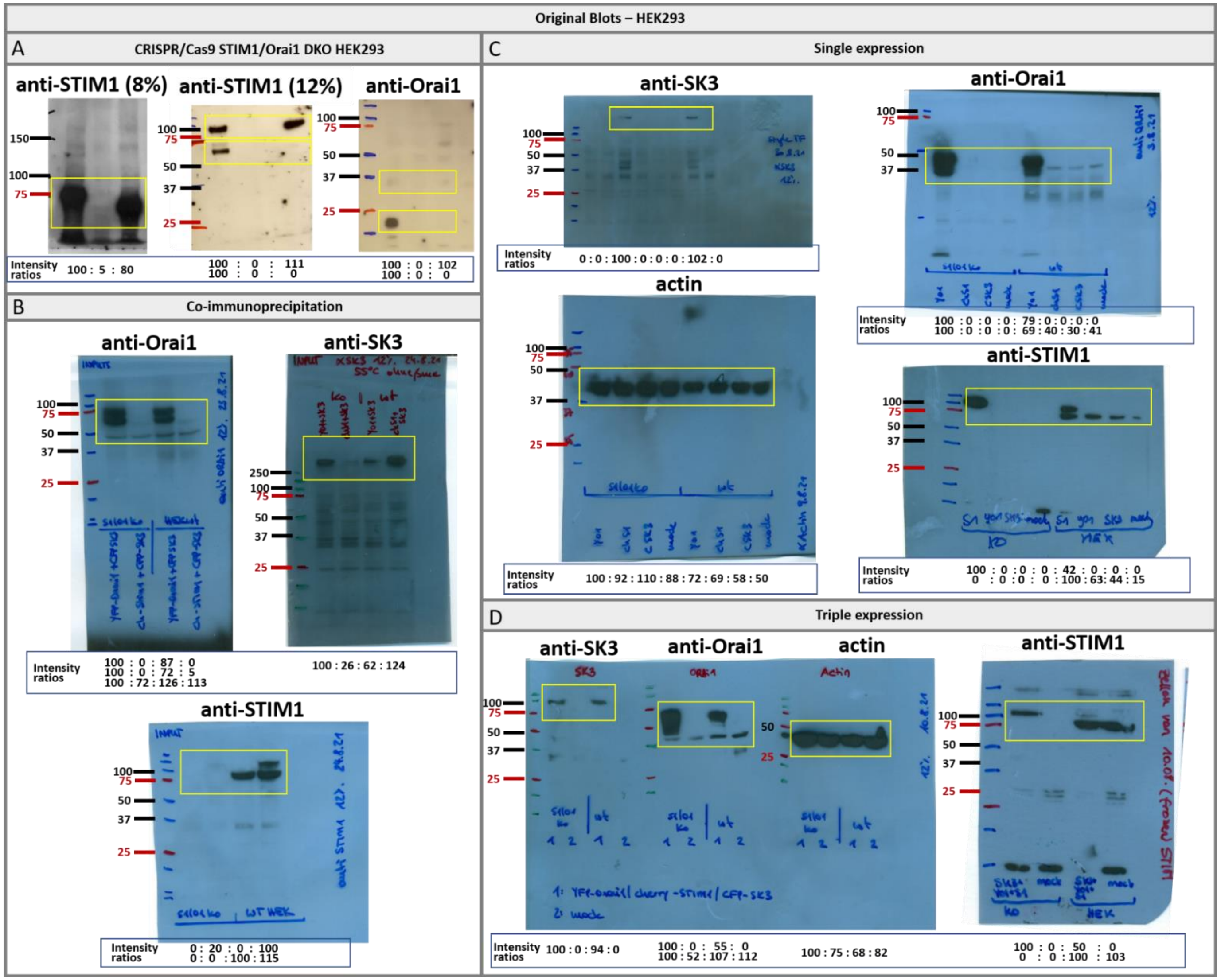

Figure S5. Original Western Blots of HEK293 cells. (**A**) Western blots corresponding to Figure S4 (A). (**B**) Western blots corresponding to Figure 2 (J) and in addition Western blot anti-STIM1 with 12% SDS. (**C**) Western blots corresponding to Figure S4 (C). (**D**) Western blots corresponding Figure S4 (D). Intensity ratios (intensities (arbitrary units) were obtained by ImageJ) of essential bands in the area of the yellow rectangle are provided on the bottom of each blot.

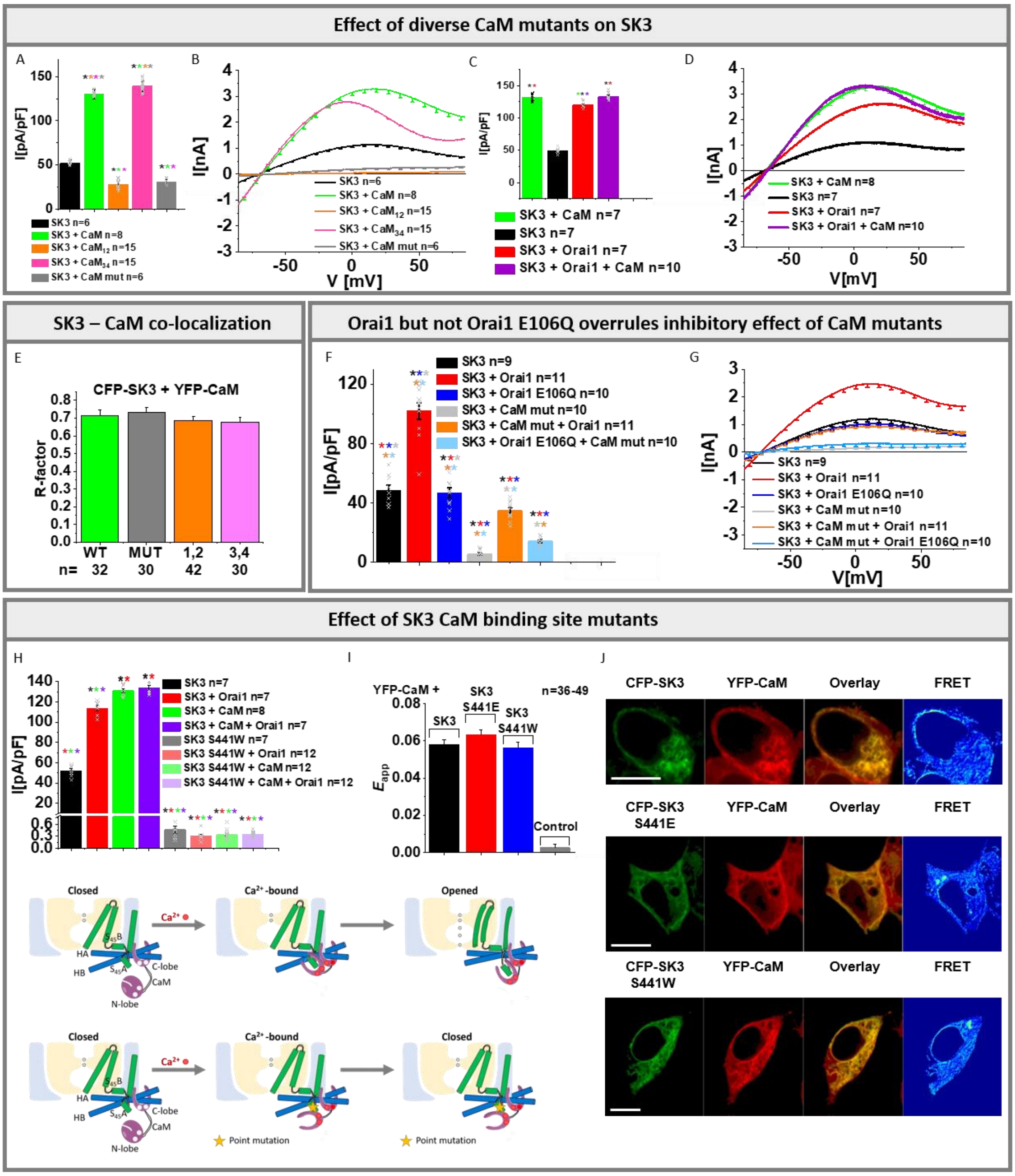

Figure S6. SK3 channel currents are regulated via CaM. (**A**) Block diagram with maximum current densities measured upon co-expression of CaM/CaM_{12}/CaM_{34}/CaM_{MUT} with SK3 channel in comparison to cells containing SK3 expressing STIM1/Orai1 DKO HEK 293 cells. The Mann-Whitney test was used for statistical comparison considering differences statistically significant at $p < 0.05$ as indicated by asterisks of the corresponding color; (**B**) I/V relationship corresponding to (**A**); (**C**) Block diagram with maximum current densities measured upon co-expression of CaM with SK3 channel in the absence or presence of Orai1 in comparison to cells containing SK3 or SK3 + Orai1 expressing STIM1/Orai1 DKO HEK 293 cells. The Mann-Whitney test was used for statistical comparison considering differences statistically significant at $p < 0.05$ as indicated by asterisks of the corresponding color; (**D**) I/V relationship corresponding to (**C**); (**E**) Co-localization of heterologously overexpressed CaM and SK3 in comparison to CaM_{12}/CaM_{34}/CaM_{MUT} and SK3; (**F**) Block diagram with maximum current densities measured upon co-expression of CaM_{MUT} with SK3 +/- Orai1 or Orai1 E106Q channel in comparison to cells containing SK3 and SK3 + Orai1 expressing STIM1/Orai1 DKO HEK 293 cells. The Mann-Whitney test was used for statistical comparison considering differences statistically significant at $p < 0.05$ as indicated by asterisks of the corresponding color; (**G**) I/V relationship corresponding to (**F**); (**H**) Block diagram with maximum current densities measured upon expression of SK3 S441W, SK3 S441W + Orai1, SK3 S441W + CaM and SK3 S441W + CaM + Orai1 compared to SK3, SK3 + Orai1, SK3 + CaM and SK3 + CaM + Orai1 in STIM1/Orai1 DKO HEK 293 cells. The Mann-Whitney test was used for statistical comparison considering differences statistically significant at $p < 0.05$ as indicated by asterisks of the corresponding color; (**I**) FRET of heterologously overexpressed YFP-CaM and CFP-SK3 S441E or CFP-SK3 S441W in comparison to YFP-CaM and CFP-SK3 and control YFP-CaM with CFP; (**J**) Co-localization and FRET of YFP-CaM with CFP-SK3 S441W or CFP-SK3 S441W in the cell membrane of STIM1/Orai1 DKO HEK 293 cells compared to YFP-CaM with CFP-SK3

(scale bar: 10 µm); FRET of heterologously overexpressed CaM and SK3 S441E or SK3 S441W in comparison to CaM and SK3. The scheme represents the proposed structure of the single subunit of the SK channel with constitutively bound CaM. The subunit consists of 6 TM domains with the pore region located between the 5th and 6th segments. The opening mechanism of the channel is illustrated. Upon SK3 CaM point mutation the channel remains in the closed state. All experiments in Figure S6 were performed in STIM1/Orai1 DKO HEK293 cells.

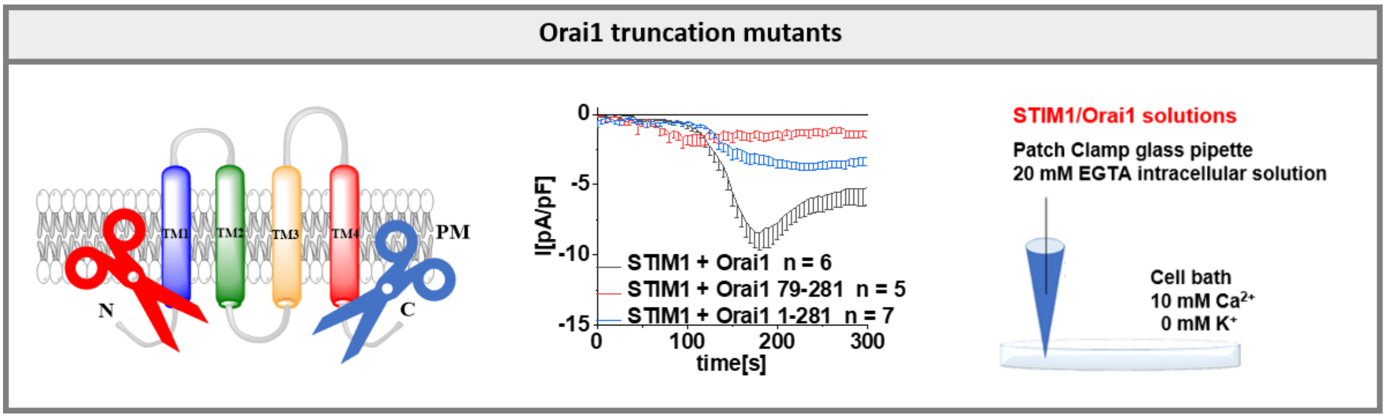

Figure S7. STIM1-mediated activation of Orai1 deletion mutants. The scheme indicates the deletion of the Orai1 N- and/or C-termini. Time course of whole-cell inward currents at -74 mV of the Orai1 fragments 79-281 and Orai1 1-281 in co-expression with STIM1 compared to Orai1 + STIM1. Inward currents activated upon passive store-depletion via 20 mM EGTA are shown in 10 mM extracellular Ca^{2+} solution as indicated on the right. All experiments in Figure S7 were performed in STIM1/Orai1 DKO HEK293 cells.

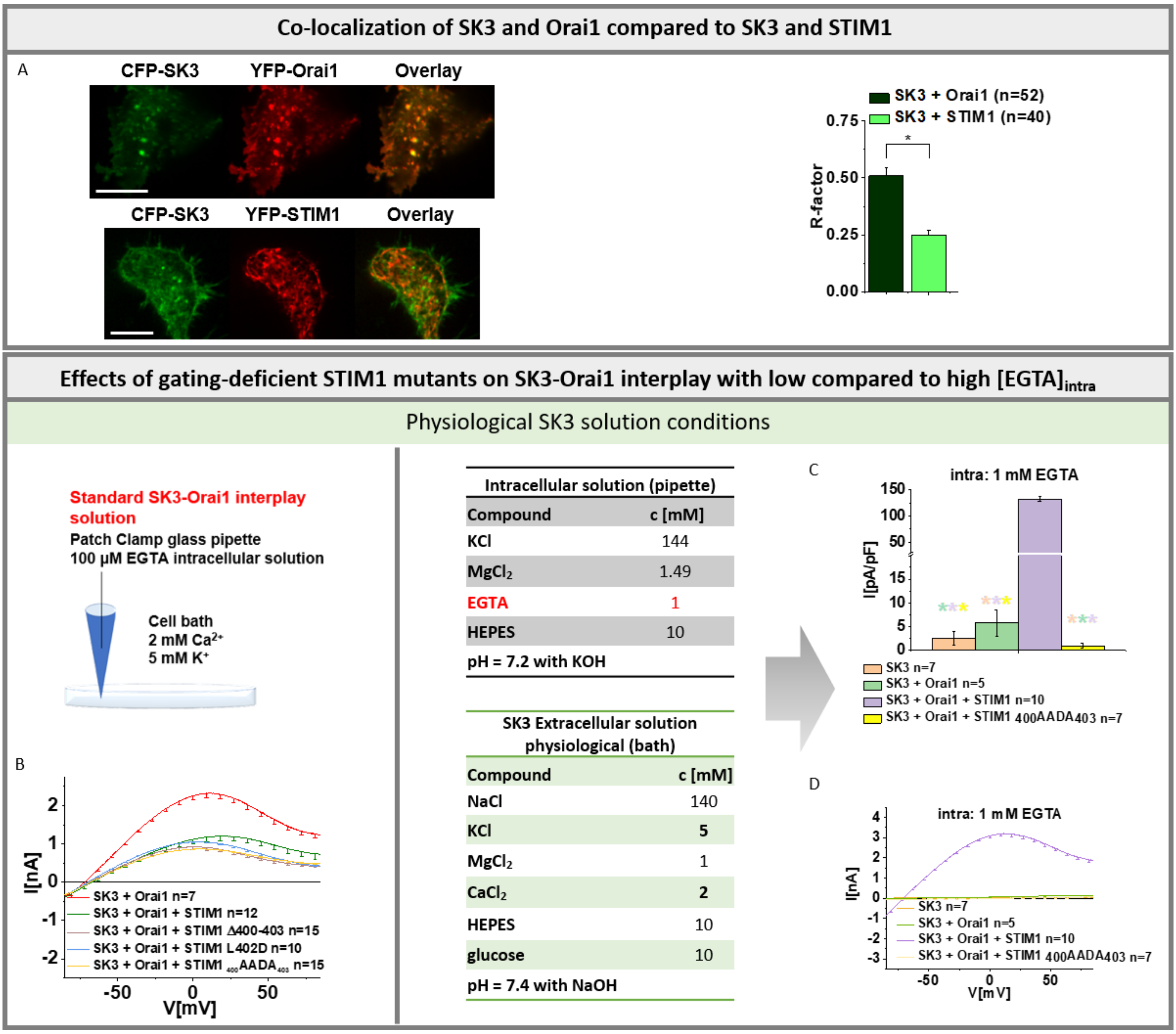

Figure S8. SK3-Orai1 and STIM1. (**A**) Co-localization studies in STIM1/Orai1 DKO HEK 293 cells performed with a pixel-by-pixel analysis and the corresponding block diagram showing the comparison of YFP-SK3 with CFP-Orai1 and CFP-SK3 with YFP-STIM1. YFP-SK3 with CFP-Orai1 displayed significantly higher co-localization than YFP-SK3 with CFP-STIM1 (scale bar: 10 µm). An asterisk indicates a significant difference in co-localization (t-test: $p < 0.05$). **(B)** I/V relationship measured in STIM1/Orai1 DKO HEK 293 cells co-expressing SK3 + Orai1 + STIM1Δ400-403/L402D/$_{400}$AADA$_{403}$ mutants in comparison to cells containing SK3 + Orai1 + STIM1 upon application of physiological SK3 solution conditions with $[EGTA]_{intra}$ of 100 µM as also indicated by the scheme on the top of the graph. (**C**) Block diagram with maximum current densities measured upon co-expression of SK3 + Orai1 + STIM1 $_{400}$AADA$_{403}$ in comparison to SK3, SK3 + Orai1 and SK3 + Orai1 + STIM1 expressing STIM1/Orai1 DKO HEK 293 cells upon application of standard physiological solution conditions with higher $[EGTA]_{intra}$ of 1mM as indicated by the tables in the middle. The Mann-Whitney test was used for statistical comparison considering differences statistically significant at $p < 0.05$ as indicated by asterisks of the corresponding color; (**D**) I/V relationship corresponding to (c). All experiments in Figure S8 were performed in STIM1/Orai1 DKO HEK293 cells.

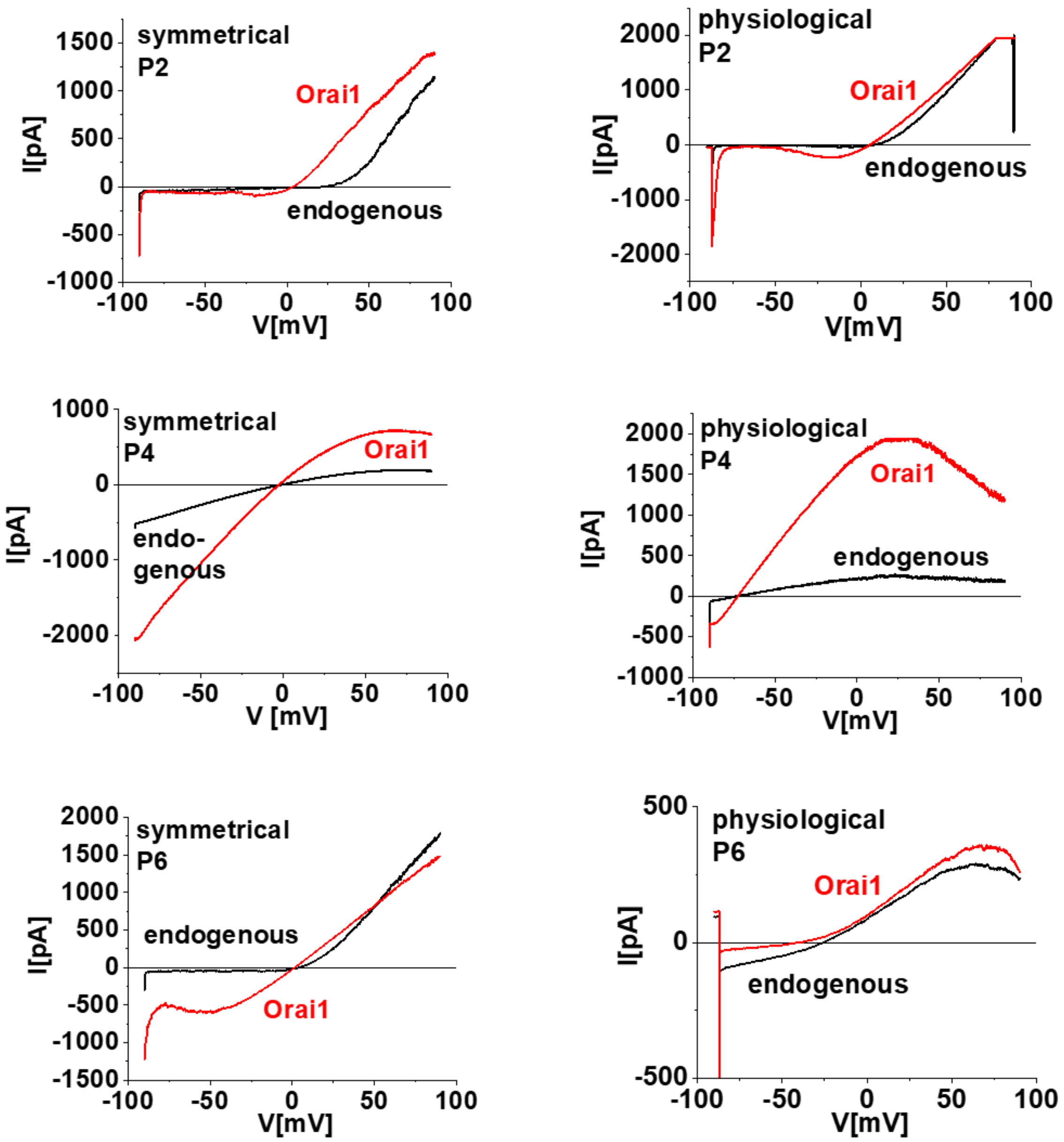

Figure S9. I/V relationships of K^+ currents in LNCaP cells. Comparison of I/V traces of K^+ currents of untransfected versus Orai1 overexpressing LNCaP cells of different passages P2, P4 and P6. Currents are shown under symmetrical (left) and physiological (right) solution conditions.

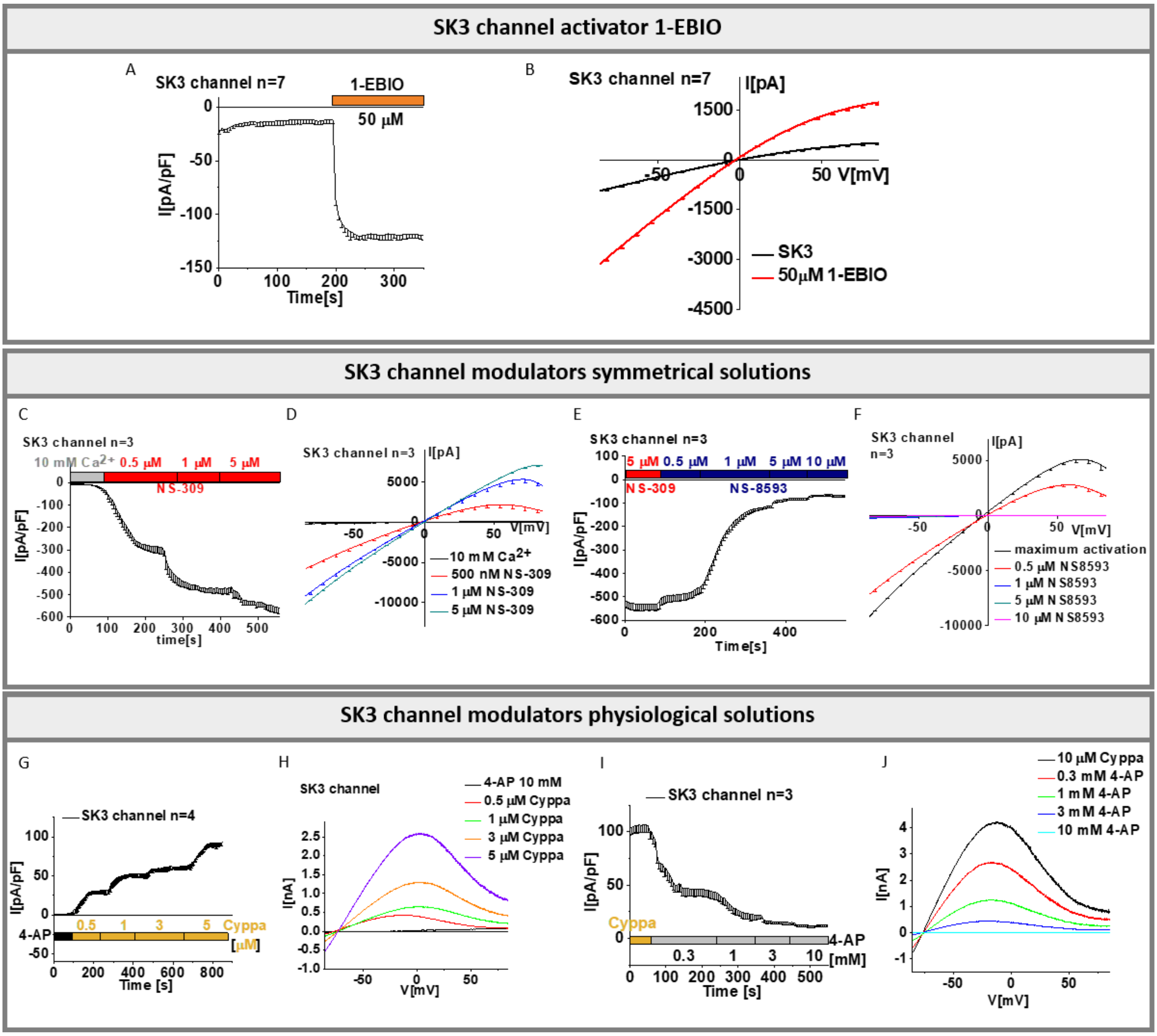

Figure S10. Effects of SK channel agonists & antagonists on SK3 channel currents. (**A**) Time course of SK3 channel before and after application of agonist 50 μM 1-EBIO; (**B**) I/V relationship corresponding to (**A**); (**C**) Time course of SK3 channel after application of agonist NS309. NS309 [0.5; 1; 5 μM] was perfused to the bath solution as indicated by the bars; (**D**) I/V relationship corresponding to (**C**); (**E**) Time course of maximally activated SK3 channel after application of agonist NS309 and subsequent inactivation by antagonist NS8593. NS8593 [0.5; 1; 5; 10 μM] was perfused to the bath solution as indicated by the bars; (**F**) I/V relationship corresponding to (**E**); (**G**) Time course of SK3 channel after application of agonist Cyppa. Cyppa [0.5; 1; 3; 5 μM] was perfused to the bath solution as indicated by the bars; (**H**) I/V relationship corresponding to (**G**); (**I**) Time course of maximally activated SK3 channel after application of agonist Cyppa and subsequent inactivation by antagonist 4-AP. 4-AP [0.3; 1; 3; 10 mM] was perfused to the bath solution as indicated by the bars; (**J**) I/V relationship corresponding to (**I**). All experiments in Figure S5 were performed in normal HEK 293 cells.

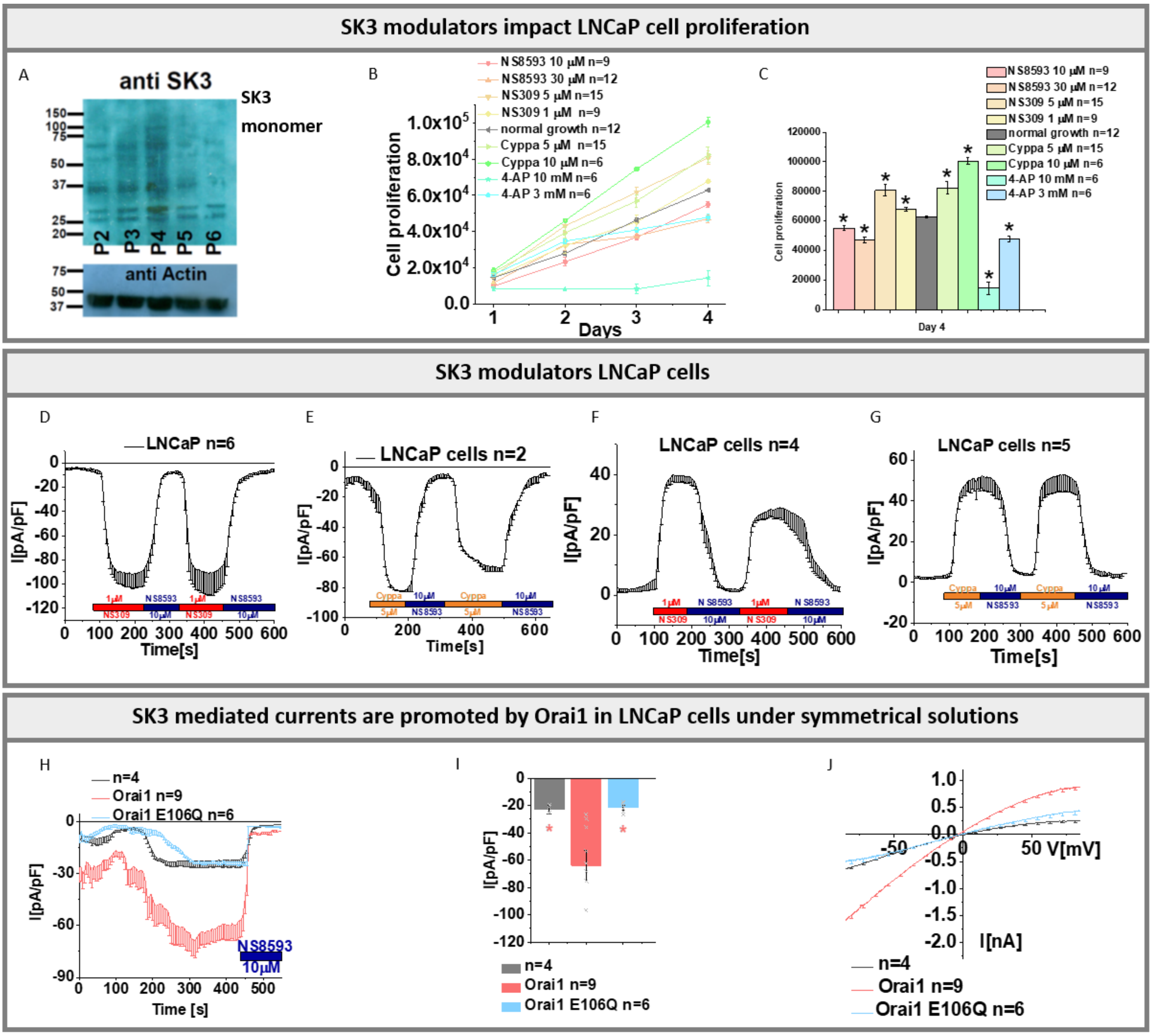

Figure S11. SK3 channel is endogenously expressed in LNCaP cells. (**A**) Western blot showing SK3 expression in LNCaP cells of different passages: P2, P3, P4, P5 and P6. (**B**) Cell viability of LNCaP cells after 24/48/72 and 96 h upon the treatment with SK3 channel agonists 5 µM, 10 µM Cyppa; 1 µM, 5 µM, NS309 and antagonists 10 µM, 30 µM NS8593; 3 mM, 10 mM 4-AP detected via MTS assay; (**C**) Block diagram represents the cell proliferation of LNCaP cells upon conditions described in (**A**). The Mann-Whitney test was used for statistical comparison considering differences statistically significant at $p < 0.05$, the black asterisk represents the significance in relation to normal cell growth; (**D**, **E**) Time course of untransfected LNCaP cells after application of agonist 1 µM NS309 (**D**) or 5 µM Cyppa (**E**) and subsequent inactivation by antagonist 10 µM NS8593 under symmetrical solution conditions. The treatment of 1 µM NS309 (**D**) or 5 µM Cyppa (**E**) and subsequent inhibition by antagonist 10 µM NS8593 was reversible and displayed upon second application comparable behavior like after first treatment; (**F**, **G**) The same experimental design as indicated in (**D**, **E**) recorded under physiological solution conditions; (**H**) Time course of LNCaP currents of endogenously expressed SK3 channel in the absence or presence of Orai1 or Orai1 E106Q under symmetrical solution conditions. (**I**) Block diagram with maximum current densities corresponding to (**H**); The Mann-Whitney test was used for statistical comparison considering differences statistically significant at $p < 0.05$ as indicated by asterisks of corresponding color;(**J**) I/V relationship of maximum currents measured in (**H**). All experiments in Figure S9 were performed in LNCaP cells.

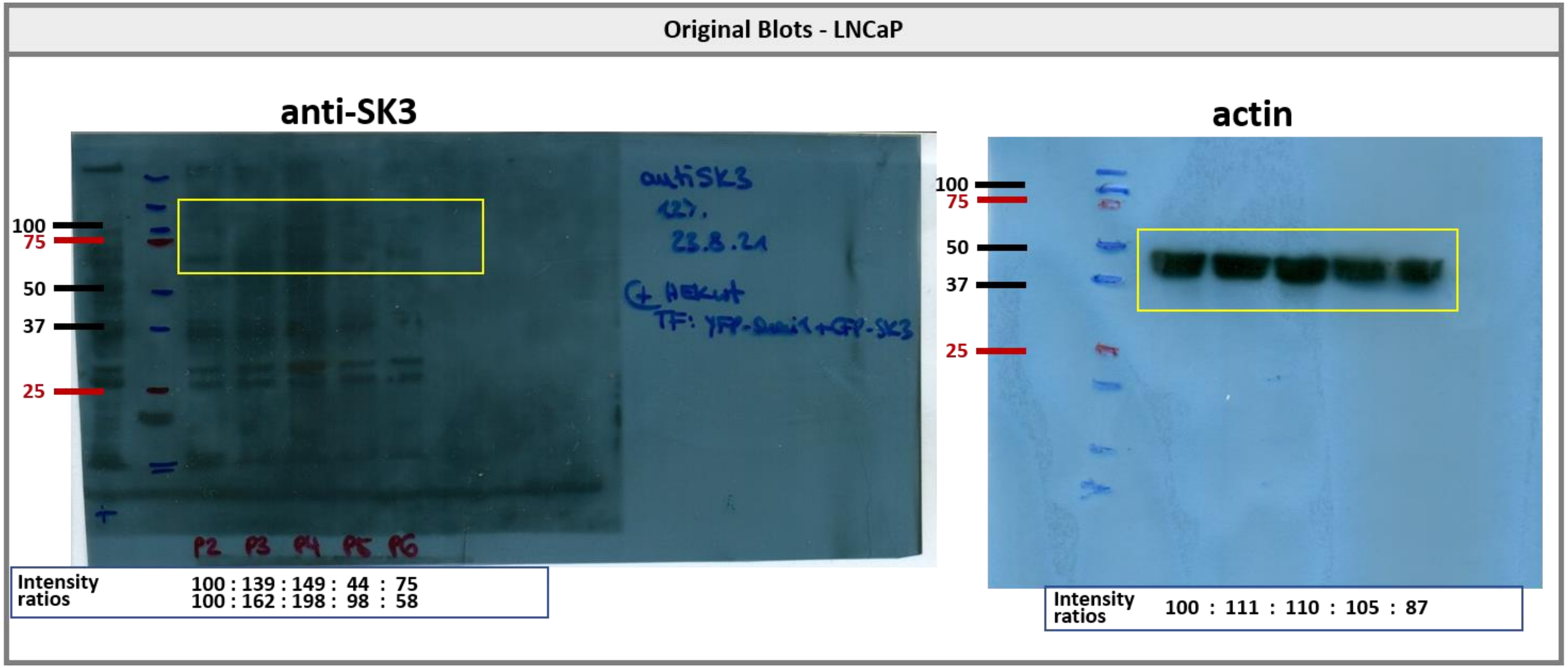

Figure S12. Original Western blots of LNCaP cells. Western blots corresponding to Figure S11 (A) showing SK3 expression in LNCaP cells of different passages: P2, P3, P4, P5 and P6. Intensity ratios (intensities (arbitrary units) were obtained by ImageJ) of essential bands in the area of the yellow rectangle are provided on the bottom of each blot.

Publisher's Note: MDPI stays neutral with regard to jurisdictional claims in published maps and institutional affiliations.

© 2021 by the authors. Licensee MDPI, Basel, Switzerland. This article is an open access article distributed under the terms and conditions of the Creative Commons Attribution (CC BY) license (http://creativecommons.org/licenses/by/4.0/).
